# Combination therapy of cancer with cancer vaccine and immune checkpoint inhibitors: A mathematical model

**DOI:** 10.1371/journal.pone.0178479

**Published:** 2017-05-25

**Authors:** Xiulan Lai, Avner Friedman

**Affiliations:** 1 Institute for Mathematical Sciences, Renmin University of China, Beijing, P. R. China; 2 Mathematical Biosciences Institute & Department of Mathematics, Ohio State University, Columbus, OH, United States of America; University of Queensland Diamantina Institute, AUSTRALIA

## Abstract

In this paper we consider a combination therapy of cancer. One drug is a vaccine which activates dendritic cells so that they induce more T cells to infiltrate the tumor. The other drug is a checkpoint inhibitor, which enables the T cells to remain active against the cancer cells. The two drugs are positively correlated in the sense that an increase in the amount of each drug results in a reduction in the tumor volume. We consider the question whether a treatment with combination of the two drugs at certain levels is preferable to a treatment by one of the drugs alone at ‘roughly’ twice the dosage level; if that is the case, then we say that there is a positive ‘synergy’ for this combination of dosages. To address this question, we develop a mathematical model using a system of partial differential equations. The variables include dendritic and cancer cells, CD4^+^ and CD8^+^ T cells, IL-12 and IL-2, GM-CSF produced by the vaccine, and a T cell checkpoint inhibitor associated with PD-1. We use the model to explore the efficacy of the two drugs, separately and in combination, and compare the simulations with data from mouse experiments. We next introduce the concept of synergy between the drugs and develop a synergy map which suggests in what proportion to administer the drugs in order to achieve the maximum reduction of tumor volume under the constraint of maximum tolerated dose.

## Introduction

When cancer cells undergo necrosis, they release high mobility group box-1 (HMGB-1) which activates dendritic cells [1–3]. Activated dendritic cells (DCs) mature as APC cells and play a critical role in the communication between the innate and adaptive immune responses. Once activated, dendritic cells produce IL-12, which activates effector T cells CD4^+^ Th1 and CD8^+^ T [4, 5]. Th1 produces IL-2 which further promotes proliferation of the effector T cells. Both CD4^+^ Th1 and CD8^+^ T cells kill cancer cells [6–8]. CD8^+^ T cells are more effective in killing cancer cells, but the helper function of CD4^+^ Th1 cells improves the efficacy of tumor-reactive CD8^+^ T cells [9].

Cancer vaccines serve to enlarge the pool of tumor-specific T cells from the naive repertoire, and also to activate tumor specific T cells which are dormant [10]. GM-CSF can activate dendritic cells, and is commonly used as a cancer vaccine [11–13]. GVAX is a cancer vaccine composed of tumor cells genetically modified to secrete GM-CSF and then irradiated to prevent further cell division.

PD-1 is an immunoinhibitory receptor predominantly expressed on activated T cells [14, 15]. Its ligand PD-L1 is upregulated on the same activated T cells, but it is also expressed by some human cancer cells, such as in melanoma, lung cancer, colon cancer, and leukemia [15–17]. The complex PD-1-PD-L1 is known to inhibit T cell function [14]. Immune checkpoints are regulatory pathways in the immune system that inhibit its active response against specific targets. In the case of cancer, the complex PD-1-PD-L1 forms an immune checkpoint for T cells.

There has been much progress in recent years in developing checkpoint inhibitors, primarily PD-1 antibodies and PD-L1 antibodies [17]. Such drugs have been increasingly explored in single-agent studies for cancer treatment [16, 18]. The FDA recently approved several checkpoint inhibitors. However, because of lack of tumor-infiltrating effector T cells, many patients in clinical trials do not respond to checkpoint inhibitor treatment [18]. On the other hand, cancer vaccines have been shown to induce effector T-cells infiltration into tumors [19], although, to be fully effective, cancer vaccines have to overcome immune evasion [10]. It was recently suggested that the combination of a cancer vaccine and an immune checkpoint inhibitor may function synergistically to induce more effective antitumor immune responses [18, 20]. Clinical trials to test such combination therapies are currently ongoing [18, 20]; mouse experiments are also being conducted [21–27].

**Fig 1:**
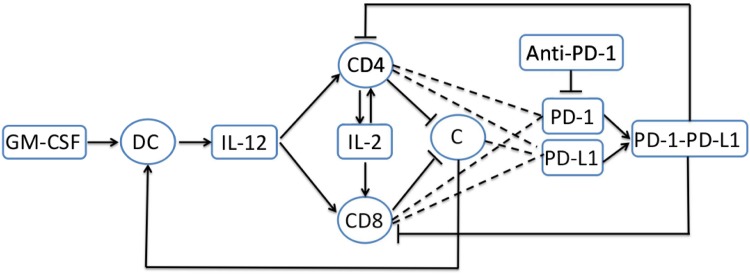
Interaction of immune cells with tumor cells. Sharp arrows indicate proliferation/activation, blocked arrows indicate killing/blocking, and dashed lines indicate proteins on T cells. GM-CSF activates dendritic cells; activated dendritic cells produce IL-12; IL-12 activates naive CD4^+^ and CD8^+^ T cells; activated CD4^+^ T cells (Th1) produce IL-2 which induces proliferation of activated CD4^+^ and CD8^+^ T cells. Activated CD4^+^ and CD8^+^ T cells kill cancer cells. Activated CD4^+^ and CD8^+^ T cells express PD-1 and PD-L1, and cancer cells express PD-L1. The complex PD-1-PD-L1 inhibits the function of active CD4^+^ and CD8^+^ T cells.

In the present paper we develop a mathematical model of treatment of cancer with a cancer vaccine combined with an immune checkpoint inhibitor; specifically, we combine GVAX and PD-1 inhibitor. In order to focus on the combination therapy of the two drugs, we consider in the model only the following variables: cancer cells (C), dendritic cells (DCs), CD4^+^ and CD8^+^ T cells, GM-CSF, PD-1, PD-L1, PD-1-PD-L1 complex, and cytokines IL-12 and IL-2. These species interact within the network shown in Fig 1. The mathematical model is based on Fig 1, and it is represented by a system of partial differential equations (PDEs). Simulations of the model are shown to be in qualitative agreement with the mouse experiments reported in [21–23]. The model is then used to explore the efficacy of the combined treatment. We introduce a specific concept of synergy between the vaccine and the PD-1 inhibitor, which is somewhat different from the usual definition of synergy. Roughly speaking, we compare the reduction in tumor size achieved by a combined therapy with amounts *γ*_*G*_ of GVAX and *γ*_*A*_ of PD-1 inhibitor to the reduction obtained by single-agent with either (1 + *θ*_*G*_)*γ*_*G*_ or (1 + *θ*_*A*_)*γ*_*A*_ with appropriately chosen 0 < *θ*_*G*_ ≤ 1 or 0 < *θ*_*A*_ ≤ 1. The larger the reduction in tumor size achieved by the combination therapy the larger the synergy is said to be. A specific choice of *θ*_*G*_ and *θ*_*A*_, which takes into account potential negative side-effects of each drug, will be given as an example. We develop a synergy map in the (*γ*_*G*_, *γ*_*A*_)-plane. The map shows that, given *γ*_*G*_, the synergy increases as *γ*_*A*_ increases as long as *γ*_*A*_ remains below a critical value *γ*_*AG*_; thereafter, the synergy decreases as *γ*_*A*_ increases.

A discrete-time mathematical model of the combination of radiotherapy with checkpoint inhibitors was developed in [28]. Mathematical models of immunotherapy with a cancer vaccine by a system of ordinary differential equations were developed earlier in [29, 30]; these models do not consider checkpoint inhibitors. In [29] it is shown that there is a positive correlation between the number of antigen presenting cells and prolonged cancer dormancy, and in [30] it is illustrated how the combined therapy can eliminate the cancer, though neither immunotherapy nor checkpoint inhibitors can do it alone. In this paper, we present for the first time a mathematical model which combines a cancer vaccine with a checkpoint inhibitor: the vaccine (GVAX) increases the pool of T cells and the checkpoint inhibitor (anti-PD-1) enables the T cells to remain fully active in killing cancer cells.

## Mathematical model

The mathematical model is based on the network shown in Fig 1. The list of variables, with units, is given in Table 1.

**Table 1:** List of variables.

Notation	Description	units
*D*	density of DCs	g/cm^3^
*T*_1_	density of activated CD4^+^ T cells	g/cm^3^
*T*_8_	density of activated CD8^+^ T cells	g/cm^3^
*C*	density of cancer cells	g/cm^3^
*N*_*C*_	density of necrotic cancer cells	g/cm^3^
*H*	HMGB-1 concentration	g/cm^3^
*G*	GM-CSF concentration	g/cm^3^
*I*_12_	IL-12 concentration	g/cm^3^
*I*_2_	IL-2 concentration	g/cm^3^
*P*	PD-1 concentration	g/cm^3^
*L*	PD-L1 concentration	g/cm^3^
*Q*	PD-1-PD-L1 concentration	g/cm^3^
*A*	anti-PD-1 concentration	g/cm^3^

We assume that the total density of cancer cells (*C*), active dendritic cells (*D*), *CD*4^+^ T cells (*T*_1_) and *CD*8^+^ T cells (*T*_8_) within the tumor remains constant in space and time:

$$\begin{array}{c} C+D+T_{1}+T_{8}=\;\text{constant}. \end{array}$$ (1)

It is tacitly assumed that the debris of dead cells, including cancer cells undergoing necrosis or apoptosis, is quickly cleared from the tumor tissue. It is also tacitly assumed that the densities of immature dendritic cells and naive CD4^+^ and CD8^+^ T cells are constant throughout the tumor tissue.

Under the assumption Eq (1), cancer cell proliferation, and migration of immune cells into the tumor give rise to internal pressure which results in cell movement, and we assume that all the cells move with the same velocity, **u**; **u** depends on space and time. We also assume that all the cells undergo diffusion, and that all the cytokines and drugs are diffusing within the tumor.

**Equation for DCs (*D*).** When cancer cells undergo necrosis, they release HMGB-1 [1]. We can model the dynamics of the necrotic cells and of HMGB-1 by the following equations:

$$\begin{array}{ccc} & & \frac{\partial N_{C}}{\partial t}+\underset{\text{velocity}}{\underset{︸}{\nabla \cdot (uN_{C})}}-\underset{\text{difusion}}{\underset{︸}{\delta_{N_{C}}\nabla^{2}N_{C}}}=\underset{\text{derived}\;\text{from}\;\text{life}\;\text{cancer}\;\text{cells}}{\underset{︸}{\lambda_{N_{C}C}C}}-\underset{\text{removal}}{\underset{︸}{d_{N_{C}}N_{C}}},\\ & & \frac{\partial H}{\partial t}-\underset{\text{difusion}}{\underset{︸}{\delta_{H}\nabla^{2}H}}=\underset{\text{released}\;\text{from}\;\text{nerotic}\;\text{cancer}\;\text{cells}}{\underset{︸}{\lambda_{HN_{C}}N_{C}}}-\underset{\text{degradation}}{\underset{︸}{d_{H}H}}, \end{array}$$

where *λ*_*N*_*C*_*C*_ is the rate at which cancer cells become necrotic and *λ*_*HN*_*C*__ is the rate at which necrotic cells produce HMGB-1. We note that although molecules like HMGB-1, or other proteins, may be affected by the velocity u, their diffusion coefficients are several orders of magnitude larger than the diffusion coefficients of cells; hence their velocity terms may be neglected. The degradation of HMGB-1 is fast (∼0.01/day) [31], and we assume that the removal of *N*_*C*_ is also fast. We can therefore approximate the two dynamical equations by the steady state equations *λ*_*N*_*C*_*C*_*C* − *d*_*N*_*C*__*N*_*C*_ = 0 and *λ*_*HN*_*C*__*N*_*C*_ − *d*_*H*_*H* = 0, so that *H* is proportional to *C*, i.e., *H* = constant × *C*.

Dendritic cells are activated by HMGB-1 [2, 3]. Hence, the activation rate of immature dendritic cell *D*_0_ is proportional to $D_{0}\frac{C}{K_{C}+C}$, where the Michaelis-Menten law is used to account for the limited rate of receptor recycling time which occurs in the process of DCs activation. In the same way, GM-CSF, produced by the cancer vaccine, activates DCs at rate proportional to $D_{0}\frac{G}{K_{G}+G}$. Hence, the dynamics of DCs is given by

$$\begin{array}{c} \frac{\partial D}{\partial t}+\underset{\text{velocity}}{\underset{︸}{\nabla \cdot (uD)}}-\underset{\text{difusion}}{\underset{︸}{\delta_{D}\nabla^{2}D}}=\underset{\text{activation}\;\text{by}\;\text{HMGB-1}}{\underset{︸}{\lambda_{DC}D_{0}\frac{C}{K_{C}+C}}}+\underset{\text{promotion}\;\text{by}\;\text{GM-CSF}}{\underset{︸}{\lambda_{DG}D_{0}\frac{G}{K_{G}+G}}}-\underset{\text{death}}{\underset{︸}{d_{D}D}}, \end{array}$$ (2)

where *δ*_*D*_ is the diffusion coefficient and *d*_*D*_ is the death rate of DCs.

**Equation for CD4^+^ T cells (*T*_1_).** Naive CD4^+^ T cells are activated by IL-12, and IL-2 induces proliferation of activated *T*_1_ cells [4, 5]. Both processes are assumed to be inhibited by the complex PD-1-PD-L1 (*Q*) [14], which reduces the production of *T*_1_ cells by a factor $\frac{1}{1+Q/K_{TQ}}$. Hence *T*_1_ satisfies the following equation:

$$\begin{array}{ccc} \frac{\partial T_{1}}{\partial t}+\nabla \cdot \left(uT_{1}\right)-\delta_{T}\nabla^{2}T_{1} & = & \underset{\text{activation}\;\text{by}\;\text{IL-12}}{\underset{︸}{(\lambda_{T_{1}I_{12}}T_{10}\frac{I_{12}}{K_{I_{12}}+I_{12}}}}+\underset{\text{IL-2-induced}\;\text{proliferation}}{\underset{︸}{\lambda_{T_{1}I_{2}}T_{1}\frac{I_{2}}{K_{I_{2}}+I_{2}})}}\\ & & \times \underset{\text{inhibition}\;\text{by}\;\text{PD-1-PD-L1}}{\underset{︸}{\frac{1}{1+Q/K_{TQ}}}}-\underset{\text{death}}{\underset{︸}{d_{T_{1}}T_{1}}}, \end{array}$$ (3)

where *T*_10_ is the density of naive CD4^+^ T cells.

**Equation for activated CD8^+^ T cells (*T*_8_).** IL-12 activates CD8^+^ T cells and IL-2 induces the proliferation of CD8^+^ T cells [4, 5]. Hence, similarly to the equation for *T*_1_, *T*_8_ satisfies the equation

$$\begin{array}{ccc} \frac{\partial T_{8}}{\partial t}+\nabla \cdot \left(uT_{8}\right)-\delta_{T}\nabla^{2}T_{8} & = & \underset{\text{activation}\;\text{by}\;\text{IL-12}}{\underset{︸}{(\lambda_{T_{8}I_{12}}T_{80}\frac{I_{12}}{K_{I_{12}}+I_{12}}}}+\underset{\text{IL-2-induced}\;\text{proliferation}}{\underset{︸}{\lambda_{T_{8}I_{2}}T_{8}\frac{I_{2}}{K_{I_{2}}+I_{2}})}}\\ & & \times \underset{\text{inhibition}\;\text{by}\;\text{PD-1-PD-L1}}{\underset{︸}{\frac{1}{1+Q/K_{TQ}}}}-\underset{\text{death}}{\underset{︸}{d_{T_{8}}T_{8}}}, \end{array}$$ (4)

where *T*_80_ is the density of naive CD8^+^ T cells.

**Equation for tumor cells (*C*).** Cancer cells are killed by *T*_1_ and *T*_8_ [6–8]. We assume a logistic growth with carrying capacity (*C*_*M*_) in order to account for competition for space among the cancer cells. Hence,

$$\begin{array}{ccc} \frac{\partial C}{\partial t}+\nabla \cdot \left(uC\right)-\delta_{C}\nabla^{2}C & = & \underset{\text{proliferation}}{\underset{︸}{\lambda_{C}C(1-\frac{C}{C_{M}})}}-\underset{\text{killing}\;\text{by}\;\text{T}\;\text{cells}}{\underset{︸}{\left(\eta_{1}T_{1}C+\eta_{8}T_{8}C\right)}}-\underset{\text{death}}{\underset{︸}{d_{C}C}}, \end{array}$$ (5)

where *η*_1_, *η*_8_ are the killing rates of cancer cells by *T*_1_ and *T*_8_, and *d*_*C*_ is the natural death rate of cancer cells.

**Equation for GM-CSF (*G*).** We assume that GVAX is injected intradermally every 3 days for 30 days (as in mouse experiments [21]) providing a source $\hat{G}\left(t\right)$ of GM-CSF, which we represent by

$$\begin{array}{c} \hat{G}\left(t\right)=\{\begin{array}{cc} \gamma_{G} & \;\text{if}\;t\leq 30,\\ \gamma_{G}\times \frac{33-t}{3} & \;\text{if}\;30<t\leq 33,\\ 0 & \;\text{if}\;t>33. \end{array} \end{array}$$

where *γ*_*G*_ is the effective level of the drug; although the level of the drug varies between injections, for simplicity we take it to be constant. The concentration of GM-CSF in tissue is very small [32], and accordingly, we assume a low rate of constant source *λ*_*G*_ for GM-CSF. Hence *G* satisfies the following equation:

$$\begin{array}{ccc} \frac{\partial G}{\partial t}-\delta_{G}\nabla^{2}G & = & \lambda_{G}+\hat{G}\left(t\right)-\underset{\text{degradation}}{\underset{︸}{d_{G}G}}, \end{array}$$ (6)

where *d*_*G*_ is the degradation rate of GM-CSF.

**Equation for IL-12 (*I*_12_).** IL-12 is produced by activated DCs [4, 5], so that

$$\begin{array}{ccc} \frac{\partial I_{12}}{\partial t}-\delta_{I_{12}}\nabla^{2}I_{12} & = & \underset{\text{production}\;\text{by}\;\text{DCs}}{\underset{︸}{\lambda_{I_{12}D}D}}-\underset{\text{degradation}}{\underset{︸}{d_{I_{12}}I_{12}}}. \end{array}$$ (7)

**Equation for IL-2 (*I*_2_).** IL-2 is produced by activated CD4^+^ T cells (*T*_1_) [4, 5]. Hence,

$$\begin{array}{ccc} \frac{\partial I_{2}}{\partial t}-\delta_{I_{2}}\nabla^{2}I_{2} & = & \underset{\text{production}\;\text{by}\;T_{1}}{\underset{︸}{\lambda_{I_{2}T_{1}}T_{1}}}-\underset{\text{degradation}}{\underset{︸}{d_{I_{2}}I_{2}}}. \end{array}$$ (8)

**Equation for PD-1 (*P*), PD-L1 (*L*) and PD-1-PD-L1 (*Q*).** PD-1 is expressed on the surface of activated CD4^+^ T cells and activated CD8^+^ T cells [14, 15]. We assume that the expression level of PD-1 is the same for activated CD4^+^ and CD8^+^ T cells. Hence, *P* is given by

$$\begin{array}{c} P=\rho_{P}\left(T_{1}+T_{8}\right), \end{array}$$

where *ρ*_*P*_ is the ratio between the mass of one PD-1 protein to the mass of one T cell. The coefficient *ρ*_*P*_ is constant when no anti-PD-1 drug is injected. In that case, to a change in *T* = *T*_1_ + *T*_8_, given by $\frac{\partial T}{\partial t}$, there corresponds a change of *P*, given by $\rho_{P}\frac{\partial T}{\partial t}$. For the same reason, $\nabla \cdot \left(uP\right)=\rho_{P}\nabla \cdot \left(uT\right)$ and ∇^2^*P* = *ρ*_*P*_∇^2^*T* when no anti-PD-1 drug is injected. Hence, *P* satisfies the equation

$$\begin{array}{c} \frac{\partial P}{\partial t}+\nabla \cdot \left(uP\right)-\delta_{T}\nabla^{2}P=\rho_{P}[\frac{\partial (T_{1}+T_{8})}{\partial t}+\nabla \cdot \left(u\left(T_{1}+T_{8}\right)\right)-\delta_{T}\nabla^{2}\left(T_{1}+T_{8}\right)]. \end{array}$$

Recalling Eqs (3) and (4) for *T*_1_ and *T*_8_, we get

$$\begin{array}{ccc} \frac{\partial P}{\partial t}+\nabla \cdot \left(uP\right)-\delta_{T}\nabla^{2}P & = & \rho_{P}[\left(\lambda_{T_{1}I_{12}}T_{10}+\lambda_{T_{8}I_{12}}T_{80}\right)\frac{I_{12}}{K_{I_{12}}+I_{12}}\\ & & \;+\left(\lambda_{T_{1}I_{2}}T_{1}+\lambda_{T_{8}I_{2}}T_{8}\right)\frac{I_{2}}{K_{I_{2}}+I_{2}}]\times \frac{1}{1+Q/K_{TQ}}\\ & & -\rho_{P}\left(d_{T_{1}}T_{1}+d_{T_{8}}T_{8}\right). \end{array}$$

When anti-PD-1 drug (*A*) is applied, PD-1 is depleted (or blocked) by *A*. In this case, the ratio $\frac{P}{T_{1}+T_{8}}$ may change. In order to include in the model both cases of with and without anti-PD-1, we replace *ρ*_*P*_ in the previous equation by $\frac{P}{T_{1}+T_{8}}$. Hence,

$$\begin{array}{ccc} \frac{\partial P}{\partial t}+\nabla \cdot \left(uP\right)-\delta_{T}\nabla^{2}P & = & \frac{P}{T_{1}+T_{8}}[\left(\lambda_{T_{1}I_{12}}T_{10}+\lambda_{T_{8}I_{12}}T_{80}\right)\frac{I_{12}}{K_{I_{12}}+I_{12}}\\ & & +\left(\lambda_{T_{1}I_{2}}T_{1}+\lambda_{T_{8}I_{2}}T_{8}\right)\frac{I_{2}}{K_{I_{2}}+I_{2}}]\times \frac{1}{1+Q/K_{TQ}}\\ & & -\frac{P}{T_{1}+T_{8}}\left(d_{T_{1}}T_{1}+d_{T_{8}}T_{8}\right)-\underset{\text{depletion}\;\text{by}\;\text{anti-PD-1}}{\underset{︸}{\mu_{PA}PA,}} \end{array}$$ (9)

where *μ*_*PA*_ represents the rate at which *P* is depleted/blocked by *A*.

PD-L1 is expressed on the surface of activated CD4^+^ and CD8^+^ T cells [14, 15] and on cancer cells [15, 16]. Hence, the concentration of PD-L1 (*L*) is proportional to (*T*_1_ + *T*_8_) and *C*:

$$\begin{array}{c} L=\rho_{L}\left(T_{1}+T_{8}+\varepsilon C\right), \end{array}$$ (10)

where *ε* depends on the specific type of tumor.

PD-L1 from T cells or cancer cells ligands to PD-1 on the plasma membrane of T cells, thus forming a complex PD-1-PD-L1 (*Q*) on the T cells [15, 16]. Denoting the association and disassociation rates of *Q* by *α*_*PL*_ and *d*_*Q*_, respectively, we can write

$$\begin{array}{c} P+L\overset{\alpha_{PL}}{\underset{d_{Q}}{⇌}}Q, \end{array}$$ (11)

so that

$$\begin{array}{c} \frac{\partial Q}{\partial t}+\nabla \cdot \left(uQ\right)-\delta_{T}\nabla^{2}Q=\alpha_{PL}PL-d_{Q}Q. \end{array}$$

The half-life of *Q* is less then 1 second (i.e. 1.16 × 10^−5^ day) [33], and hence *d*_*Q*_ is very large, and we may approximate the dynamical equation by the steady state equation, *α*_*PL*_*PL* = *d*_*Q*_*Q*, or

$$\begin{array}{c} Q=\sigma PL, \end{array}$$ (12)

where *σ* = *α*_*PL*_/*d*_*Q*_.

**Equation for anti-PD-1 (*A*).** We assume that anti-PD-1 is injected intradermally every 3 days for 30 days (as in mouse experiments [21]) providing a source $\hat{A}\left(t\right)$ of anti-PD-1:

$$\begin{array}{c} \hat{A}\left(t\right)=\{\begin{array}{cc} \gamma_{A} & \;\text{if}\;t\leq 30,\\ \gamma_{A}\times \frac{33-t}{3} & \;\text{if}\;30<t\leq 33,\\ 0 & \;\text{if}\;t>33. \end{array} \end{array}$$

where *γ*_*A*_ is the effective level of the drug; although the level of the drug varies between injections, for simplicity we take it to be constant. The drug *A* is depleted in the process of blocking PD-1. Hence,

$$\begin{array}{ccc} \frac{\partial A}{\partial t}-\delta_{A}\nabla^{2}A & = & \hat{A}\left(t\right)-\underset{\text{depletion}\;\text{through}\;\text{blocking}\;\text{PD-1}}{\underset{︸}{\mu_{PA}PA}}-\underset{\text{degradation}}{\underset{︸}{d_{A}A}}, \end{array}$$ (13)

where *μ*_*PA*_ is the rate at which *A* is degraded in the process of blocking PD-1.

**Equation for cell velocity (u)**: We assume that most of the tumor consists of the extracellular matrix, ECM (approximately, 0.6 g/cm^3^), and cancer cells (approximately, *C* = 0.4 g/cm^3^), and that the densities of *D*, *T*_1_ and *T*_8_ are approximately 4 × 10^−4^, 2 × 10^−3^ and 1 × 10^−3^ g/cm^3^, respectively (as explained in the section on parameter estimation). We further assume that all cells are approximately of the same volume and surface area, so that their diffusion coefficients are the same. For definiteness, we take the constant in Eq (1) to be 0.4034. Adding Eqs (2)–(5), we then get

$$\begin{array}{c} 0.4034\times \nabla \cdot u=\sum_{j=2}^{5}[\text{RHS}\;\text{of}\;\text{Eq.}\;(j)]. \end{array}$$ (14)

To simplify the computations, we shall assume that the tumor is spherical and denote its moving boundary (i.e. its radius) by *r* = *R*(*t*). We also assume that all the densities and concentrations are radially symmetric, that is, functions of (*r*, *t*), where 0 ≤ *r* ≤ *R*(*t*). In particular, $u=u\left(r,t\right)e_{r}$, where $e_{r}$ is the unit radial vector, and each of the equations of the form

$$\begin{array}{c} \frac{\partial X}{\partial t}+\nabla \cdot \left(uX\right)-\delta_{X}\nabla^{2}X=F, \end{array}$$

with *X* = *X*(*r*, *t*), takes the form

$$\begin{array}{c} \frac{\partial X}{\partial t}+\frac{1}{r^{2}}\frac{\partial }{\partial r}\left(r^{2}uX\right)-\delta_{X}\frac{1}{r^{2}}\frac{\partial }{\partial r}(r^{2}\frac{\partial X}{\partial r})=F. \end{array}$$

**Equation for free boundary (*R*)**: We assume that the free boundary *r* = *R*(*t*) moves with the velocity of cells, so that

$$\begin{array}{c} \frac{dR(t)}{dt}=u\left(R\left(t\right),t\right). \end{array}$$ (15)

**Boundary conditions** We assume that the naive CD4^+^ T cells and naive CD8^+^ T cells which migrated from the lymph nodes into the tumor microenvironment have constant densities ${\hat{T}}_{1}$ and ${\hat{T}}_{8}$ at the tumor boundary, and that they are activated by IL-12 upon entering the tumor. We represent this process by the flux conditions at the boundary *r* = *R*(*t*):

$$\begin{array}{c} \frac{\partial T_{1}}{\partial r}+\sigma_{T}\left(I_{12}\right)\left(T_{1}-{\hat{T}}_{1}\right)=0,\;\frac{\partial T_{8}}{\partial r}+\sigma_{T}\left(I_{12}\right)\left(T_{8}-{\hat{T}}_{8}\right)=0, \end{array}$$ (16)

where $\sigma_{T}\left(I_{12}\right)=\sigma_{0}\frac{I_{12}}{I_{12}+K_{I_{12}}}$.

We impose the no-flux boundary conditions for all the remaining variables:

$$\begin{array}{c} \text{No-flux}\;\text{for}\;D\left(r,t\right),C\left(r,t\right),G\left(r,t\right),I_{12}\left(r,t\right),I_{2}\left(r,t\right),\\ P(r,t)\;\;\text{and}\;A(r,t),\;\;\text{at}\;r=R(t). \end{array}$$ (17)

It is tacitly assumed here that the receptors PD-1 and ligands PD-L1 become active only after the T cells are inside the tumor.

**Initial conditions.** We take initial values of all the variables to be constant. Later on we shall compare the simulations of the model with mouse experimental results, for 60 days. Accordingly, we take initial values whereby the average density of cancer cells has not yet increased to its steady state, 0.4 g/cm^3^, and, in view of Eq (1), the total density of the immune cells is initially above its steady state. We take (in unit of g/cm^3^):

$$\begin{array}{c} D=6\times 10^{-4},\;T_{1}=4\times 10^{-3},\;T_{8}=2\times 10^{-3},\;C=0.3968. \end{array}$$ (18)

We assume that initially *A* = 0, and

$$\begin{array}{ccc} G & = & 2.61\times 10^{-10}\;\;\text{g}/\;{\text{cm}}^{3},\;I_{12}=1.8\times 10^{-10}\;\;\text{g}/\;{\text{cm}}^{3},\;I_{2}=4.74\times 10^{-11}\;\;\text{g}/\;{\text{cm}}^{3},\\ P & = & 11.2\times 10^{-10}\;\;\text{g}/\;{\text{cm}}^{3}. \end{array}$$

These values are close to the steady state values which are computed in the section on parameter estimation.

## Results

The simulations of the model were performed by Matlab based on the moving mesh method for solving partial differential equations with free boundary [34] (see the section on computational method). All the computations are done in dimensionless form, but displayed in dimensional form.

**Fig 2:**
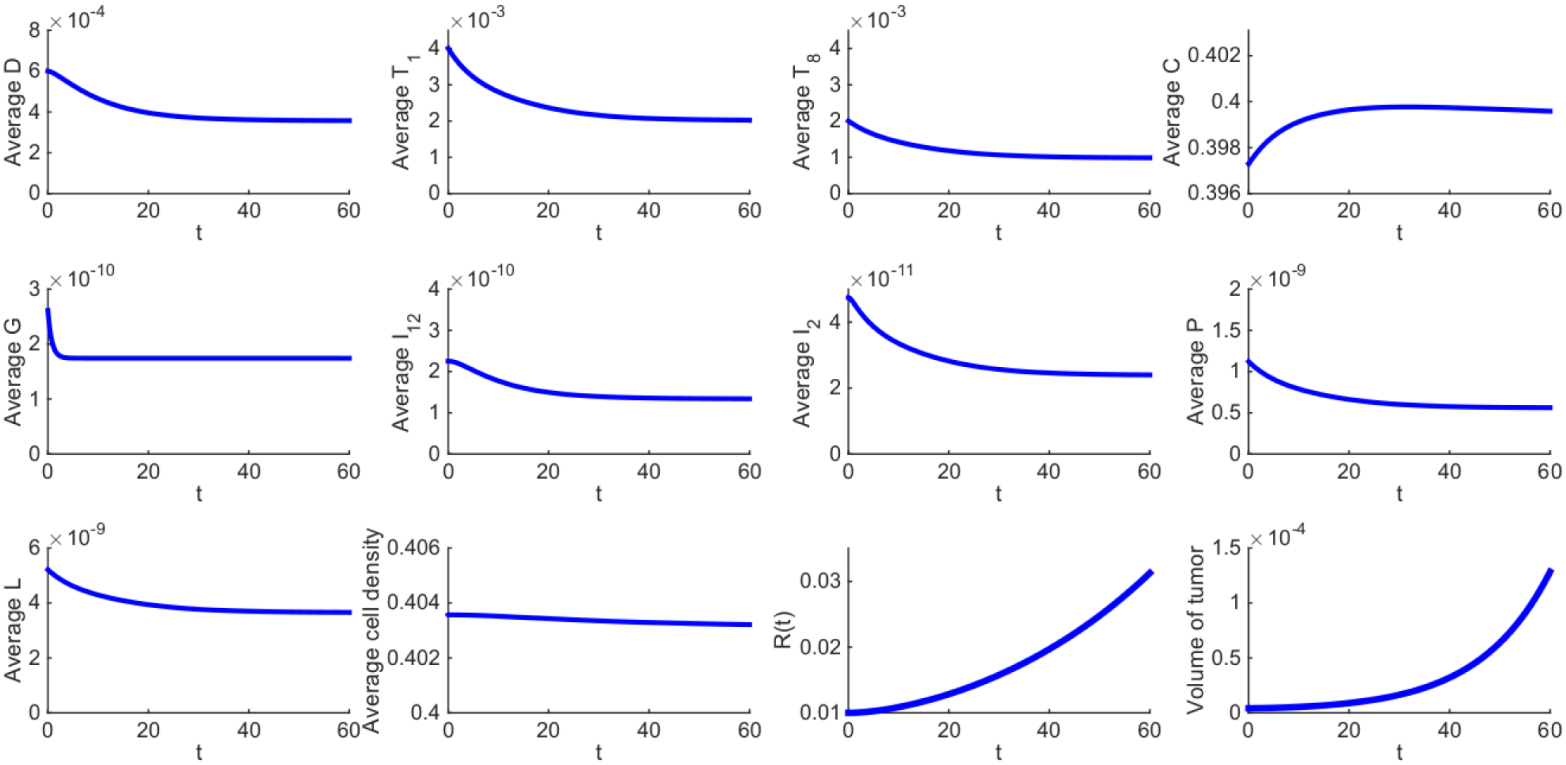
Average densities/concentrations of all the variables in the model in the control case (no drugs). Figures in the first and second panels and the first figure in the third panel show the average density or concentration of each species in the model. The last three figures in the third panel show the total average density of all cells, the growth of tumor radius and the growth of tumor volume, respectively. All parameter values are the same as in Tables 2 and 3.

The average density or concentration of a species is defined as its total mass in the tumor divided by the tumor volume. Fig 2 shows the average concentrations of all the species of the model over a period of 60 days in the control case, that is, when no drugs are administered; the parameter values are given in Tables 2 and 3. The radius of the tumor is increasing, from 0.01 cm to 0.0313 cm. The average density of the cancer cells is initially increasing and later it stabilizes while the densities of the immune cells are first decreasing and later stabilize. Correspondingly, the concentrations of the cytokines produced by the immune system also first decrease and later stabilize. Some of the parameters in the model were estimated by assuming the immune cells and cytokines are in steady state. Their steady states in Fig 2 approximately agree with those which we assumed in estimating the parameters, thus establishing the consistency of our assumed steady-state values.

**Table 2:** Summary of parameter values.

Notation	Description	Value used	References
*δ*_*D*_	diffusion coefficient of DCs	8.64 × 10^−7^ cm^2^day^−1^	[59]
*δ*_*T*_1__	diffusion coefficient of CD4^+^ T cells	8.64 × 10^−7^ cm^2^day^−1^	[59]
*δ*_*T*_8__	diffusion coefficient of CD8^+^ T cells	8.64 × 10^−7^ cm^2^day^−1^	[59]
*δ*_*C*_	diffusion coefficient of tumor cells	8.64 × 10^−7^ cm^2^day^−1^	[59]
*δ*_*G*_	diffusion coefficient of GM-CSF	9.8495 × 10^−2^ cm^2^day^−1^	estimated
*δ*_*I*_12__	diffusion coefficient of IL-12	6.0472 × 10^−2^ cm^2^day^−1^	estimated
*δ*_*I*_2__	diffusion coefficient of IL-2	9.9956 × 10^−2^ cm^2^day^−1^	estimated
*δ*_*A*_	diffusion coefficient of anti-PD-1	7.85 × 10^−2^ cm^2^day^−1^	estimated
*σ*_0_	flux rate of T cells on the boundary	1 cm^−1^	[59]
*λ*_*DG*_	activation rate of DCs by GM-CSF	20.02 day^−1^	[65, 66] & estimated
*λ*_*DC*_	activation rate of DCs by tumor cells	0.364 day^−1^	estimated
*λ*_*T*_1_*I*_12__	activation rate of CD4^+^ T cells by IL-12	4.66 day^−1^	estimated
*λ*_*T*_1_*I*_2__	activation rate of CD4^+^ T cells by IL-2	0.25 day^−1^	estimated
*λ*_*T*_8_*I*_12__	activation rate of CD8^+^ T cells by IL-12	4.15 day^−1^	estimated
*λ*_*T*_8_*I*_2__	activation rate of CD8^+^ T cells by IL-2	0.25 day^−1^	[59]
*λ*_*C*_	growth rate of cancer cells	0.616 day^−1^	[59]
*λ*_*G*_	non-vaccine source of GM-CSF	2.23 × 10^−10^ g/cm^3^ ⋅ day	estimated
*λ*_*I*_12_*D*_	production rate of IL-12 by DCs	5.18 × 10^−7^ day^−1^	estimated
*λ*_*I*_2_*T*_1__	production rate of IL-2 by CD4^+^ T cells	2.82 × 10^−8^ day^−1^	estimated
*η*_1_	killing rate of tumor cells by CD4^+^ T cells	11.5 day^−1^ ⋅ cm^3^/g	estmated
*η*_8_	killing rate of tumor cells by CD8^+^ T cells	46 day^−1^ ⋅ cm^3^/g	estimated
*μ*_*PA*_	blocking rate of PD-1 by anti-PD-1	6.87 × 10^6^ cm^3^/g ⋅ day	estimated
*ρ*_*P*_	expression of PD-1 in T cells	2.49 × 10^−7^	estimated
*ρ*_*L*_	expression of PD-L1 in T cells	5.22 × 10^−7^	estimated
*ε*	expression of PD-L1 in tumor cells	0 − 0.01*	estimated
*d*_*D*_	death rate of DCs	0.1 day^−1^	[59]
*d*_*T*_1__	death rate of CD4^+^ T cells	0.197 day^−1^	[59]
*d*_*T*_8__	death rate of CD8^+^ T cells	0.18 day^−1^	[59]
*d*_*C*_	death rate of tumor cells	0.17 day^−1^	[59]
*d*_*G*_	degradation rate of GM-CSF	1.28 day^−1^	[65]
*d*_*I*_12__	degradation rate of IL-12	1.38 day^−1^	[59]
*d*_*I*_2__	degradation rate of IL-2	2.376 day^−1^	[59]
*d*_*A*_	degradation rate of anti-PD-1	0.0462 day^−1^	[42]

* In the simulations we took *ε* = 0.01.

**Table 3:** Summary of parameter values.

*K*_*G*_	half-saturation of GM-CSF	1.74 × 10^−9^ g/cm^3^	[65]
*K*_*C*_	half-saturation of tumor cells	0.4 g/cm^3^	[59]
*K*_*I*_12__	half-saturation of IL-12	1.5 × 10^−10^ g/cm^3^	[59]
*K*_*I*_2__	half-saturation of IL-2	2.37 × 10^−11^ g/cm^3^	[59]
*K*_*T*_1__	half-saturation of CD4^+^ T cells	2 × 10^−3^ g/cm^3^	estimated
*K*_*T*_8__	half-saturation of CD8^+^ T cells	1 × 10^−3^ g/cm^3^	estimated
$K_{TQ}^{\prime }$	inhibition of function of T cells by PD-1-PD-L1	1.365 × 10^−18^ g/cm^3^	estimated
*D*_0_	density of immature DCs	2 × 10^−5^ g/cm^3^	[59]
*T*_10_	density of naive CD4^+^ T cells	4 × 10^−4^ g/cm^3^	estimated
*T*_80_	density of naive CD8^+^ T cells	2 × 10^−4^ g/cm^3^	estimated
*C*_*M*_	carrying capacity of cancer cells	0.8 g/cm^3^	[59]
${\hat{T}}_{1}$	density of CD4^+^ T cells from lymph node	4 × 10^−3^ g/cm^3^	estimated
${\hat{T}}_{8}$	density of CD8^+^ T cells from lymph node	2 × 10^−3^ g/cm^3^	estimated
*γ*_*G*_	source of GM-CSF from the vaccine	1 × 10^−10^ g/cm^3^ ⋅ day*	estimated
*γ*_*A*_	source of anti-PD-1	1 × 10^−10^ g/cm^3^ ⋅ day*	estimated

* Values used in sensitivity analysis.

**Fig 3:**
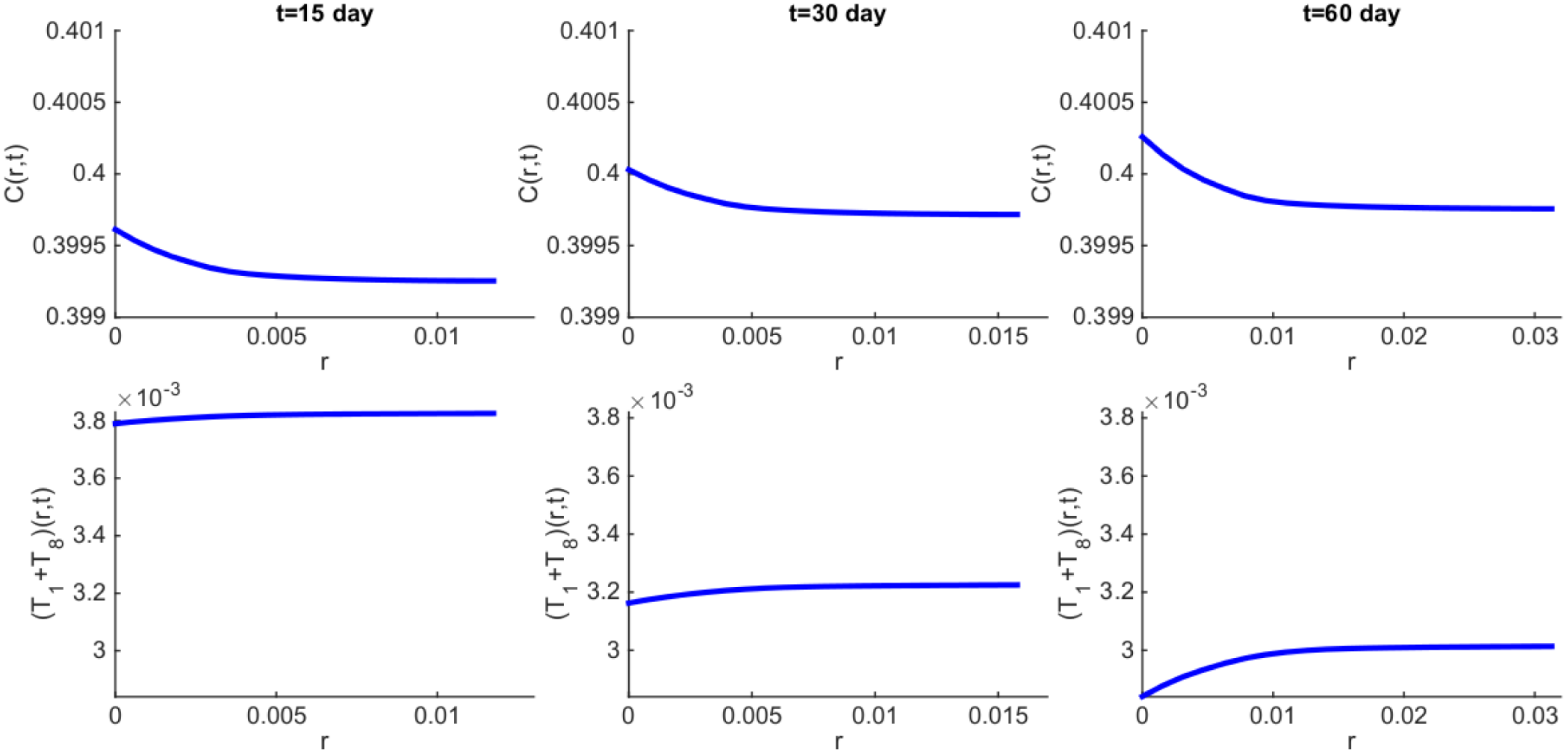
Spatial distribution of density of cancer cells and *T*_1_ + *T*_8_ cells at time *t* = 15, 30, 60 days. All parameter values are the same as in Fig 2.

We can also simulate the spatial distribution of each of the variables. Fig 3 shows the distribution of cancer cells and T cells (*T*_1_ + *T*_8_) at times *t* = 15, 30, 60 days. We see that the density of T cells increases toward the boundary; this is a result of the influx of T cells from the lymph nodes. Correspondingly, the density of cancer cells decreases toward the boundary. In our model, we tacitly assumed avascular conditions, since we wanted to focus primarily on the difference between control and treatment. Since, however, the tumor radius reaches approximately 313 *μ*m at day 60, hypoxic conditions may actually reduce the cancer cells’ density at the core of the tumor (both in control and treatment).

In Figs 2 and 3, we have taken *ε* = 0.01. Some cancer cells may express more or less PD-L1 [17, 35–37]. By decreasing or increasing *ε*, the radius of the tumor will decrease or increase, respectively, but the profiles of the densities/concentrations remain qualitatively the same (not shown here).

**Fig 4:**
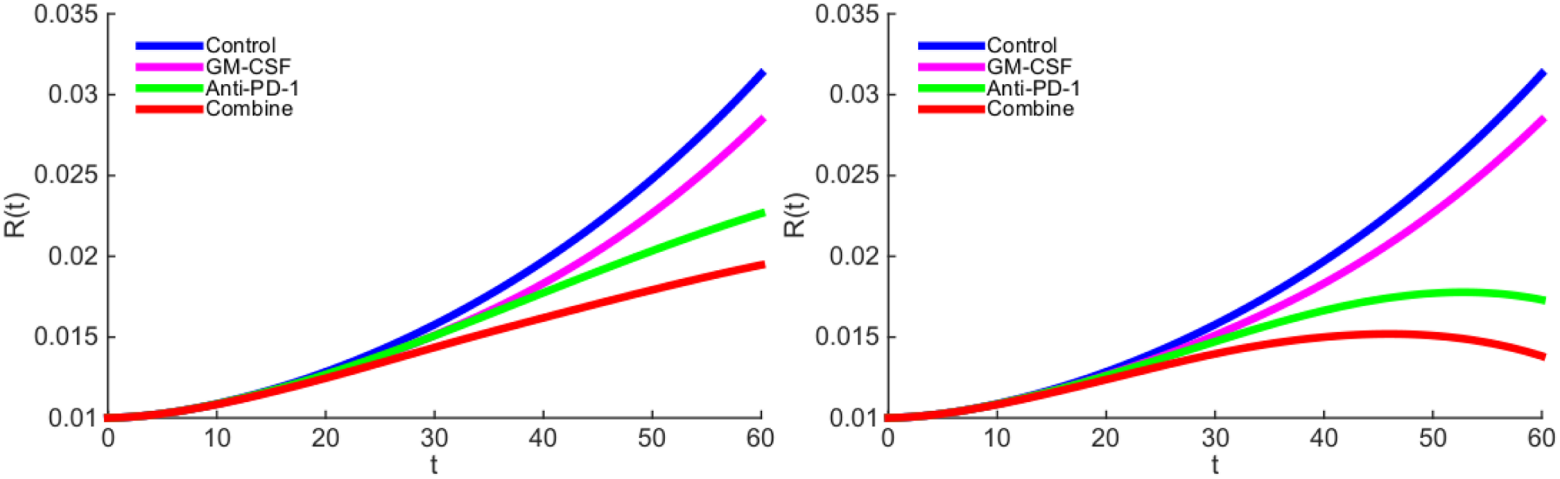
The growth of tumor radius *R*(*t*) during the administration of GM-CSF-secreting vaccine and anti-PD-1 drug. (a) GM-CSF-secreting vaccine is administered at rate *γ*_*G*_ = 0.87 × 10^−10^ g/cm^3^ ⋅ day and anti-PD-1 drug is administered at rate *γ*_*A*_ = 2 × 10^−10^ g/cm^3^ ⋅ day. (b) GM-CSF-secreting vaccine is administered at rate *γ*_*G*_ = 0.87 × 10^−10^ g/cm^3^ ⋅ day and anti-PD-1 drug is administered at rate *γ*_*A*_ = 3 × 10^−10^ g/cm^3^ ⋅ day.

We next proceed to explore the effect of treatment with GM-CSF-secreting vaccine (GVAX) and anti-PD-1 drug. We are unable to make a direct connection between the levels of drugs administered to the patient, and their ‘effective strengths’ *γ*_*G*_ and *γ*_*A*_ in the model, since these data are not available. Based on the estimate of the concentration of GM-CSF in normal healthy tissue (see Parameter Estimations for Eq (6)), we chose the order of magnitude of GVAX to be 10^−10^ (g/cm^3^ ⋅ day). The order of magnitude for anti-PD-1 drug (*γ*_*A*_) is chosen so as to get the best agreement with the mouse experiments. In Fig 4(a), the ‘effective strength’ of the vaccine is given by *γ*_*G*_ = 0.87 × 10^−10^ g/cm^3^ ⋅ day and the ‘effective strength’ of the anti-PD-1 is given by *γ*_*A*_ = 2 × 10^−10^ g/cm^3^ ⋅ day. We see that, as a single-agent, anti-PD-1 is more effective than GVAX, and with the combined therapy the tumor radius is still increasing. This is in agreement with the mouse experiments reported in Fig 1-(A) (with melanoma) in [21], and Figs 3-(b) (with colon carcinoma) and 3-(c) (with melanoma) in [22].

In Fig 4(b), we increased *γ*_*A*_ by a factor 3/2. As a result, the tumor radius begins to decrease around day 50, even when administering anti-PD-1 as single-agent. This is in agreement with mouse experiments (with colon carcinoma) reported in Fig 1-(D) of [23].

**Fig 5:**
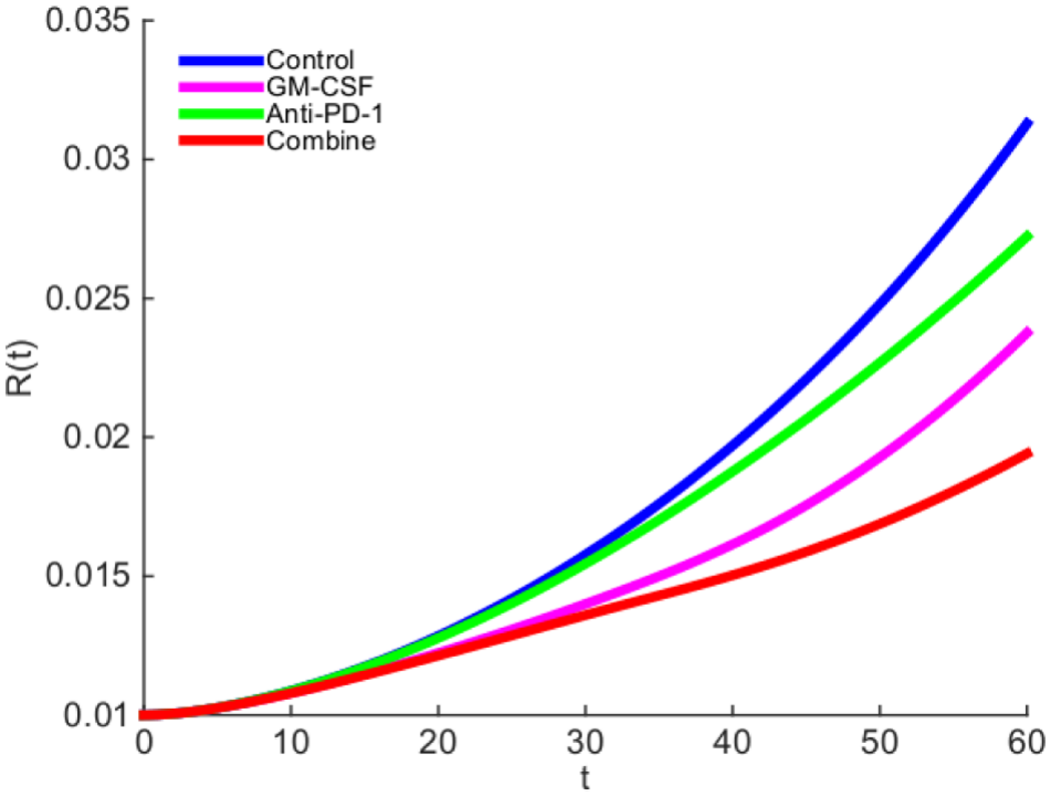
The growth of tumor radius *R*(*t*) during the administration of GM-CSF-secreting vaccine and anti-PD-1 drug. GM-CSF-secreting vaccine is administered at rate *γ*_*G*_ = 3.84 × 10^−10^ g/cm^3^ ⋅ day and anti-PD-1 drug is administered at rate *γ*_*A*_ = 1 × 10^−10^ g/cm^3^ ⋅ day.

In Fig 4(a), GM-CSF-secreting vaccine alone did not reduce the tumor radius as much as anti-PD-1 alone. However, if we increase the strength of the vaccine and decrease the anti-PD-1, taking for example, *γ*_*G*_ = 3.84 × 10^−10^ g/cm^3^ ⋅ day and *γ*_*A*_ = 1 × 10^−10^ g/cm^3^ ⋅ day, we then find that the vaccine decreases the tumor radius more than anti-PD-1 does; this is shown in Fig 5, and it is in agreement with mouse experiments (with melanoma) reported in Fig 1-(B) of [23].

The results of Figs 4 and 5 show that a combination therapy of GVAX and anti-PD-1 in appropriate amounts could significantly slow the growth of a tumor.

There is some uncertainty in the estimates of some of the parameters of the model. With somewhat different choices of these parameters, the simulation results will change quantitatively, and sensitivity analysis indicates the direction and intensity of the change (see the section on sensitivity analysis). In particular, the choice of *γ*_*G*_ and *γ*_*A*_ will affect the relative reduction in the growth of the tumor radius. In Figs 4 and 5 we made specific choices of *γ*_*G*_ and *γ*_*A*_, in order to get simulations that agree with experimental results.

**Fig 6:**
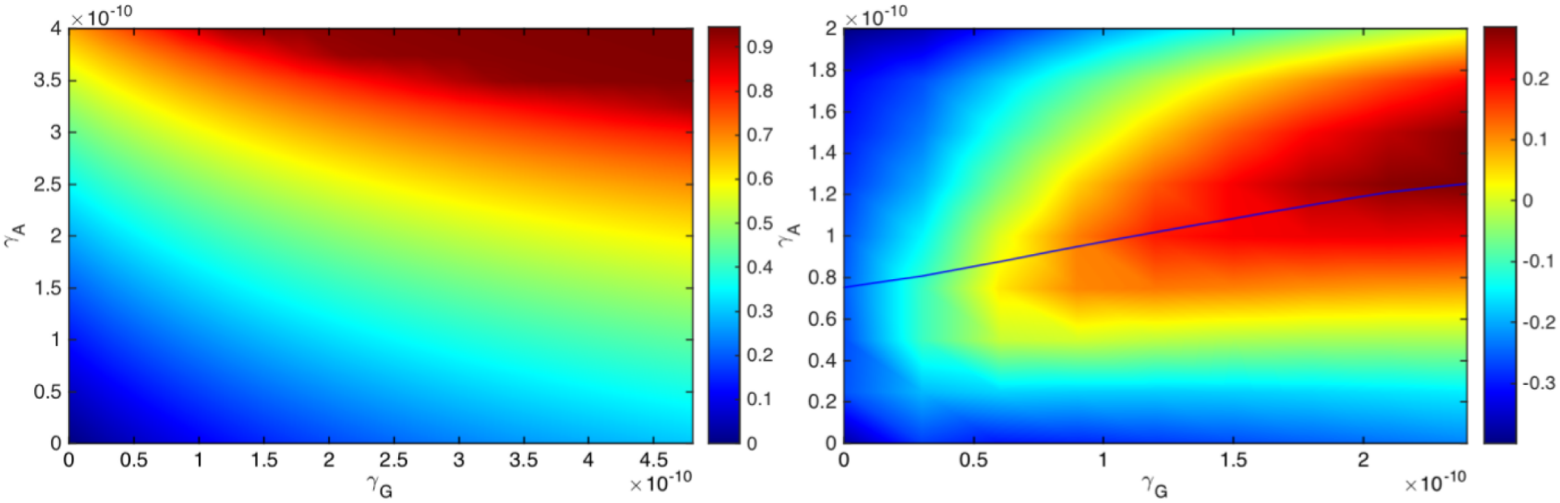
Drug efficacy map and synergy map. (a) Efficacy map: The color column shows the efficacy *E*(*γ*_*G*_, *γ*_*A*_) when *γ*_*G*_ varies between 0 − 4.8 × 10^−10^ g/cm^3^ ⋅ day and *γ*_*A*_ varies between 0 − 4 × 10^−10^ g/cm^3^ ⋅ day. (b) Synergy map: The color column shows the synergy *σ*(*γ*_*G*_, *γ*_*A*_) when *γ*_*G*_ varies between 0 − 2.4 × 10^−10^ g/cm^3^ ⋅ day and *γ*_*A*_ varies between 0 − 2 × 10^−10^ g/cm^3^ ⋅ day. For a given *γ*_*G*_, the optimal synergy of the combined therapy (*γ*_*G*_, *γ*_*A*_) occurs when (*γ*_*G*_, *γ*_*A*_) lies on the solid curve.

We next consider combination therapy for any values of GVAX and anti-PD-1. We define the efficacy of the combination therapy with (*γ*_*G*_, *γ*_*A*_) by the formula:

$$\begin{array}{c} E\left(\gamma_{G},\gamma_{A}\right)=\frac{R_{60}\left(0,0\right)-R_{60}\left(\gamma_{G},\gamma_{A}\right)}{R_{60}\left(0,0\right)}, \end{array}$$

where *R*_60_ = *R*_60_(*γ*_*G*_, *γ*_*A*_) represents the tumor radius computed at day 60. If the tumor radius at day 60, *R*_60_(*γ*_*G*_, *γ*_*A*_), is smaller than the radius in the control case, *R*_60_(0, 0), then the efficacy is a positive number, and its value is between 0 and 1 (or between 0% and 100%); the efficacy increases to 1 (or to 100%) when the tumor radius *R*_60_(*γ*_*G*_, *γ*_*A*_) decreases to 0 by day 60. Fig 6(a) is the efficacy map of the combined therapy with *γ*_*G*_ in the range of 0 − 4.8 × 10^−10^) g/cm^3^ ⋅ day and *γ*_*A*_ in the range of 0 − 4 × 10^−10^ g/cm^3^ ⋅ day. The color column in Fig 6(a) shows the efficacy for any pair of (*γ*_*G*_, *γ*_*A*_); the efficacy is positive, and its maximum is 0.95 (95%). We see that an increase in either *γ*_*G*_ or *γ*_*A*_ improves the efficacy of the treatment.

Early stage clinical trials consider the safety of each of the drugs, GVAX and anti-PD-1, separately. In the GVAX treatment of patients with pancreatic cancer, no dose-limiting toxicity or minimal toxicity was observed [38–40]. On the other hand, in treatment with anti-PD-1, mild to moderate toxicity (grades 1 and 2) was observed in melanoma [41, 42]. In non-small-cell lung cancer, 14% of the patients were observed to have more severe toxicity (grades 3 and 4) [43]. Based on these observations we conclude that anti-PD-1 causes more toxicity than GVAX. However, clinical trials on the safety and efficacy of the combined drugs are limited [23, 44, 45].

The amount of drug in clinical trials is constrained by the maximum tolerated dose (MTD). In combination therapy this constraint may depend on the proportion between the amounts of the drugs. We note that there is a large literature on the trade-off between efficacy and toxicity [46–49]. Here we consider, as an example, two treatments, $\left(\gamma_{G}^{*},\gamma_{A}^{*}\right)$ and $\left(\gamma_{G}^{**},\gamma_{A}^{**}\right)$, with $\gamma_{G}^{*}>\gamma_{G}^{**}$ and $\gamma_{A}^{*}<\gamma_{A}^{**}$, where both satisfy the MTD requirement. The question is then which of the two treatments is more effective in reducing the tumor volume. We can use the efficacy map to address such a question. We illustrate this in one special case.

We compare treatment of combination (*γ*_*G*_, *γ*_*A*_) with monotherapy GVAX and monotherapy anti-PD-1. For monotherapy with GVAX, we take (1 + *θ*_*G*_)*γ*_*G*_, and for monotherapy with anti-PD-1 we take (1 + *θ*_*A*_)*γ*_*A*_, with *θ*_*A*_ < *θ*_*G*_, to reflect the higher toxicity associated with anti-PD-1. If *E*(*γ*_*G*_, *γ*_*A*_) is larger than both *E*((1 + *θ*_*G*_)*γ*_*G*_, 0) and *E*(0, (1 + *θ*_*A*_)*γ*_*A*_), then we say that the synergy for the combination (*γ*_*G*_, *γ*_*A*_) is positive, and otherwise, we say that the synergy is negative. More generally, we define the synergy *σ* = *σ*(*γ*_*G*_, *γ*_*A*_) by the formula:

$$\begin{array}{c} \sigma \left(\gamma_{G},\gamma_{A}\right)=\frac{E(\gamma_{G},\gamma_{A})}{\;\text{max}\{E\left(\left(1+\theta_{G}\right)\gamma_{G},0\right),E\left(0,\left(1+\theta_{A}\right)\gamma_{A}\right)\}}-1. \end{array}$$ (19)

Thus *σ*(*γ*_*G*_, *γ*_*A*_) > 0 (positive synergy) if the combination (*γ*_*G*_, *γ*_*A*_) reduces tumor growth more than either one of the single agents with (1 + *θ*_*G*_)*γ*_*G*_ or (1 + *θ*_*A*_)*γ*_*A*_. Negative synergy occurs in the reverse case where instead of a combination therapy with (*γ*_*G*_, *γ*_*A*_) we achieve better reduction of the tumor radius *R*_60_ if we apply only one drug, (1 + *θ*_*G*_)*γ*_*G*_ or (1 + *θ*_*A*_)*γ*_*A*_. The above concept of synergy is somewhat different from the usual definitions of synergy. For definiteness we take *θ*_*G*_ = 1 and *γ*_*A*_ = 0.5, but this choice, which is somewhat arbitrary, could be made more precise as more clinical data become available.

Fig 6(b) is the synergy map for (*γ*_*G*_, *γ*_*A*_) in the same range as in Fig 6(a); the color column shows the synergy *σ*(*γ*_*G*_, *γ*_*A*_), with values that vary from -0.38 to 0.28.

We first note that the synergy is negative if *γ*_*G*_ < 0.2 × 10^−10^ g/cm^3^ ⋅ day. The reason is the following: if *γ*_*G*_ is small then the numbers of T cells is also small, so instead of introducing a drug *γ*_*A*_ which blocks the relatively small number of PD-1, it is more effective to increase the number of T cells by increasing *γ*_*G*_, i.e. *E*(2*γ*_*G*_, 0) > *E*(*γ*_*G*_, *γ*_*A*_).

Next, for any fixed *γ*_*G*_, as seen in Fig 6(b), the synergy first increases with *γ*_*A*_ and then decreases. In order to explain this occurrence, we note that with *γ*_*G*_ fixed, the number of T cells that arrive into the tumor microenvironment is limited, and so is the number of their PD-1. Thus, in order to block the PD-1 there is a need for only a limited amount of anti-PD-1 drug; i.e. it is ‘wasteful’ to administer too much of *γ*_*A*_. We conclude that the maximum synergy is achieved when the amount of *γ*_*A*_ is appropriately dependent on the amount of *γ*_*G*_, as indicated by the solid curve shown in Fig 6(b).

**Fig 7:**
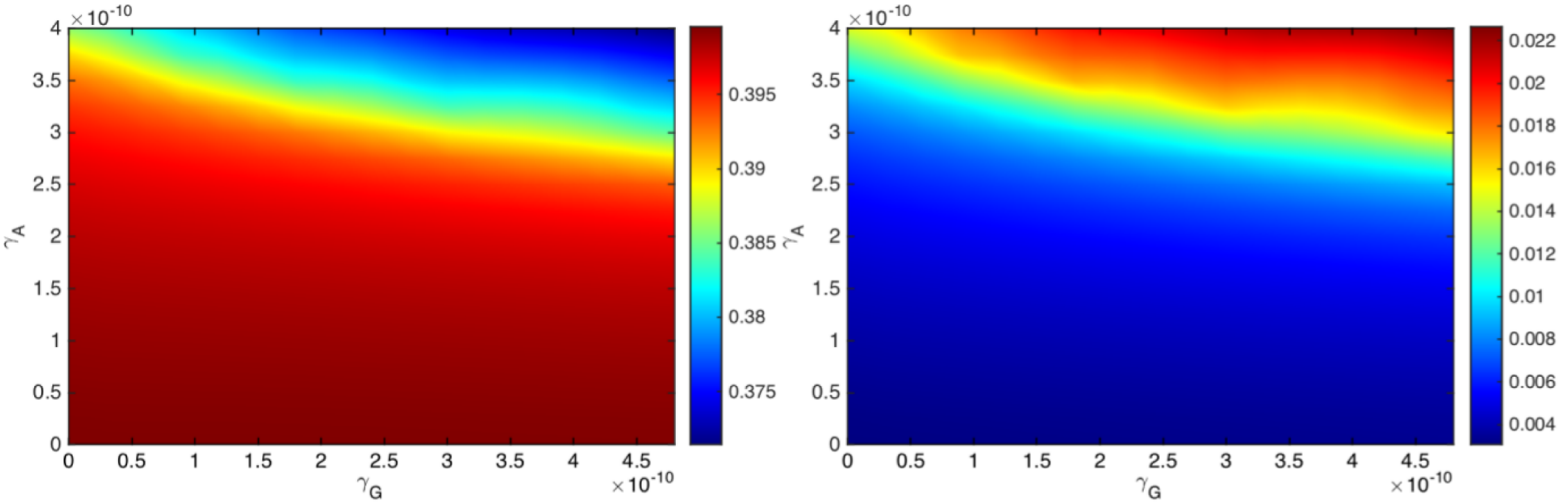
Average densities of cancer cells and T cells. (a) The average density of cancer cells (*C*), and (b) the average density of T cells (*T*_1_ + *T*_8_), under the combination of the drugs with (*γ*_*G*_, *γ*_*A*_), where *γ*_*G*_ varies between 0 − 4.8 × 10^−10^ g/cm^3^ ⋅ day and *γ*_*A*_ varies between 0 − 4 × 10^−10^ g/cm^3^ ⋅ day.

Fig 7 shows that as the amounts of *γ*_*G*_ and *γ*_*A*_ increase the average density of T cells (*T*_1_ + *T*_8_) increases, and correspondingly the average density of cancer cells decreases. We also see that the density of T cells shown in the color column increases approximately by a factor of 6, whereas the density of cancer cells decreases approximately by a factor of 1.075. The proportion of these changes (i.e. 5/0.075) is similar to the proportion of densities of cancer cells to T cells.

## Conclusion

The introduction of immune checkpoint inhibitors has been a very promising approach to cancer treatment. Blockage of the programmed death PD-1/PD-L1 is increasingly explored in single-agent studies [16, 18]. However, because of the lack of tumor-infiltrating effector T cells, many patients do not respond to checkpoint inhibitor treatment as single-agent [18]. On the other hand, cancer vaccines have been shown to induce effector T-cells infiltration into tumors [19], although, to be fully effective, cancer vaccines have to overcome immune evasion [10]. It was recently suggested that the combination of a cancer vaccine and an immune checkpoint inhibitor may function synergistically to induce more effective antitumor immune response [18, 20]. Clinical trials to test such combinations are currently ongoing [18, 20].

In the present paper we developed a mathematical model to study the efficacy of the combination of a GM-CSF-secreting cancer vaccine (GVAX) and an anti-PD-1 drug, and to address the following question: at what proportion should the two drugs be administered in order to achieve the best efficacy when the amount of drugs is limited by MTD. The mathematical model is represented by a system of partial differential equations, based on the interactions among cancer cells, dendritic cells, CD4^+^ and CD8^+^ T cells, cytokines IL-12 and IL-2, GM-CSF, PD-1, PD-L1, PD-1-PD-L1 complex and anti-PD-1. Simulations of the model are shown to be in qualitative agreement with mouse experiments [21–23].

The cancer vaccine and the anti-PD-1 work in collaboration: The vaccine increases the number of tumor-infiltrating effector T cells and the anti-PD-1 ensures that these cells remain active. Given a fixed amount *γ*_*G*_ of the vaccine and *γ*_*A*_ of the anti-PD-1, it is important to estimate the level of synergy between these amounts in order to administer them in the most effective proportion. We introduced a specific concept of synergy *σ*(*γ*_*G*_, *γ*_*A*_) and developed accordingly a synergy map in Fig 6(b). The map shows that if *γ*_*G*_ is small, single-agent treatment (with *γ*_*A*_ = 0) is the best treatment. Fig 6(b) also shows that, for any *γ*_*G*_, there is a unique value *γ*_*AG*_, such that the synergy increases as *γ*_*A*_ increases as long as *γ*_*A*_ remains smaller than *γ*_*AG*_, but the synergy decreases as *γ*_*A*_ increases above *γ*_*AG*_; the points (*γ*_*G*_, *γ*_*AG*_) form the solid curve shown in Fig 6(b). We suggest that for optimal efficacy under MTD constraint, the level of dosage of anti-PD-1 (*γ*_*A*_) should be related to the level of dosage of GVAX (*γ*_*G*_) by setting *γ*_*A*_ = *γ*_*AG*_, as indicated by the solid curve in Fig 6(b).

The mathematical model presented in this paper has several limitations:

1. In order to focus on the combined therapy of a cancer vaccine and an anti-PD-1 drug, we did not include some other important cells and cytokines that are found in the tumor microenvironment, such as T regulatory cells, macrophages, endothelial cells, and IL-10, IL-6 and TGF-β. We also did not include blood vessels and oxygen, thus assuming that the tumor is avascular. We tacitly assumed that the effect of these omissions is not significant in comparing the results of therapy to no therapy.
2. We assumed that the densities of immature, or naive, immune cells remain constant throughout the progression of the cancer and that dead cells are quickly removed from the tumor.
3. In estimating parameters we made a steady state assumption in some of the differential equations.
4. In the definition of synergy, and in the synergy map, we included in a crude way the fact that anti-PD-1 causes more toxicity than GVAX. Our aim was to develop a concept that will take account not only of efficacy but also of toxicity. For this reason we compared the treatment benefits for combination (*γ*_*G*_, *γ*_*A*_) with the single agents (2*γ*_*G*_, 0) and (0, 1.5*γ*_*A*_).
5. We did not make any direct connection between drugs administered to the patient, and their ‘effective strengths’ *γ*_*G*_ and *γ*_*A*_, as they appear in the differential equations, since these data are not available. The order of magnitude of GVAX (10^−10^) was based on the estimate of the concentration of GM-CSF in normal healthy tissue (see Parameter Estimations for Eq (6)). We simulated the model with different orders of magnitude for anti-PD-1 drug (*γ*_*A*_) and found the best agreement with the mouse experiments (in Figs 4 and 5) when *γ*_*A*_ is also of order of magnitude 10^−10^.
6. Although our mathematical model does not presume any geometric form of the tumor, for simplicity, the simulations have been carried out only in the case of a spherical tumor. We note however that spherical cancer models have been used in research as an intermediate between *in vitro* cancer line cultures and *in vivo* cancer [50]. Furthermore, spheroids mirror the 3D cellular context and therapeutically relevant pathophysiological gradient of *in vivo* tumors [51].

Clinical data on efficacy and toxicity are quite limited at this time. Our model should be viewed as setting up a computational framework, to address the question of optimal efficacy in combination therapy with cancer vaccine and checkpoint inhibitor.

## Materials and methods

### Parameter estimation

**Half-saturation**. In an expression of the form $Y\frac{X}{K_{X}+X}$ where *Y* is activated by X, the half-saturation parameter *K*_*X*_ is taken to be the approximate steady state concentration of species *X*.

**Diffusion coefficients.** By [52], we have the following relation for estimating the diffusion coefficients of a protein *p*:

$$\begin{array}{c} \delta_{p}=\frac{M_{V}^{1/3}}{M_{p}^{1/3}}\delta_{V}, \end{array}$$

where *M*_*V*_ and *δ*_*V*_ are respectively the molecular weight and diffusion coefficient of VEGF, *M*_*p*_ is the molecular weight of *p*, and *M*_*V*_ = 24kDa [53] and *δ*_*V*_ = 8.64 × 10^−2^ cm^2^day^−1^ [54]. Since *M*_*I*_2__ = 15.5kDa, *M*_*I*_12__ = 70kDa, *M*_*G*_ = 16.2kDa [55] and *M*_*A*_ = 32kDa [56], we get *δ*_*I*_2__ = 9.9956 × 10^−2^ cm^2^day^−1^, *δ*_*I*_12__ = 6.0472 × 10^−2^ cm^2^day^−1^, *δ*_*G*_ = 9.8495 × 10^−2^ cm^2^day^−1^, and *δ*_*A*_ = 7.85 × 10^−2^ cm^2^day^−1^.

Eq (2): The number of DCs in various organs (heart, kidney, pancreas and liver) in mouse varies from 1.1 × 10^6^ cells/cm^3^ to 6.6 × 10^6^ cells/cm^3^ [57]. Mature DCs are approximately 10 to 15 *μ*m in diameter [58]. Accordingly, we estimate steady states of DCs by *K*_*D*_ = 4 × 10^−4^ g/cm^3^. We assume that the density of immature DCs is smaller than the actived DCs, and take $D_{0}=\frac{1}{20}K_{D}=2\times 10^{-5}$ g/cm^3^. We also assume that the activation of DCs by GM-CSF-secreting vaccine is more effective than their activation by NCs-secreted HMGB-1, and take $\lambda_{DG}\frac{G}{K_{G}+G}=10\lambda_{DC}\frac{C}{K_{C}+C}$. From the steady state of Eq (2) in the control case (with no drug), we get

$$\begin{array}{c} \lambda_{DC}D_{0}\cdot \frac{C}{K_{C}+C}+\lambda_{DG}D_{0}\cdot \frac{G}{K_{G}+G}-d_{D}D=0, \end{array}$$

where *d*_*D*_ = 0.1/day [59], *D*_0_ = 2 × 10^−5^ g/cm^3^, *D* = *K*_*D*_ = 4 × 10^−4^ g/cm^3^, *C* = *K*_*C*_ = 0.4 g/cm^3^, and $G={\hat{K}}_{G}=1.74\times 10^{-10}$ g/cm^3^ and *K*_*G*_ = 1.74 × 10^−9^ g/cm^3^ (${\hat{K}}_{G}$ and *K*_*G*_ are estimated together with other estimates of Eq (6)). Hence *λ*_*DC*_ = 0.364/day and *λ*_*DG*_ = 20.02/day.

Eq (3): The number of lymphocytes is approximately twice the number of DCs [57]. T cells are approximately 14 to 20 *μ*m in diameter. Assuming that the number of Th1 cells is 1/4 of the number of lymphocytes, we estimate the steady state density of Th1 cells by *K*_*T*_ = 2 × 10^−3^ g/cm^3^. We assume the density of naive CD4^+^ T cells to be less than the density of Th1, and take $T_{10}=\frac{1}{5}K_{T}=4\times 10^{-4}$ g/cm^3^. We also assume that in steady state, *Q*/*K*_*TQ*_ = 2 (*K*_*TQ*_ is estimated together with other estimates of Eqs (9)–(12)). From the steady state of Eq (3), we get

$$\begin{array}{c} (\lambda_{T_{1}I_{12}}T_{10}\cdot \frac{1}{2}+\lambda_{T_{1}I_{2}}T_{1}\cdot \frac{1}{2})\cdot \frac{1}{3}-d_{T_{1}}T_{1}=0, \end{array}$$

where *T*_10_ = 4 × 10^−4^ g/cm^3^, *T*_1_ = *K*_*T*_1__ = 2 × 10^−3^ g/cm^3^, and, by [59], *λ*_*T*_1_*I*_2__ = 0.25/day and *d*_*T*_1__ = 0.197/day. Hence *λ*_*T*_1_*I*_12__ = 4.66/day.

Eq (4): The CD4/CD8 ratio in the blood is 2:1. We assume a similar ratio in the tissue, and take $T_{80}=\frac{1}{2}T_{10}=2\times 10^{-4}$ g/cm^3^. We also take the steady state of *T*_8_ to be half of the steady state of *T*_1_, i.e., $K_{T_{8}}=\frac{1}{2}K_{T_{1}}=1\times 10^{-3}$ g/cm^3^. From the steady state equation of Eq (4), we get

$$\begin{array}{c} (\lambda_{T_{8}I_{12}}T_{80}\cdot \frac{1}{2}+\lambda_{T_{8}I_{2}}T_{8}\cdot \frac{1}{2})\cdot \frac{1}{3}-d_{T_{8}}T_{8}=0, \end{array}$$

where *T*_80_ = 5 × 10^−5^ g/cm^3^, *T*_8_ = *K*_*T*_8__ = 1 × 10^−3^ g/cm^3^, and, by [59], *λ*_*T*_8_*I*_2__ = 0.25/day and *d*_*T*_8__ = 0.18/day. Hence *λ*_*T*_1_*I*_12__ = 4.15/day.

Eq (5): We take *d*_*C*_ = 0.17 day^−1^ and *C*_*M*_ = 0.8 g/cm^3^ [59]. In the absence of immune responses and anti-tumor drugs, the tumor grows according to

$$\begin{array}{c} \frac{dC}{dt}=\lambda_{C}C(1-\frac{C}{C_{M}})-d_{C}C, \end{array}$$ (20)

and with immune response, the tumor grows according to

$$\begin{array}{c} \frac{dC}{dt}=\lambda_{C}C(1-\frac{C}{C_{M}})-\left(\eta_{1}T_{1}+\eta_{8}T_{8}\right)C-d_{C}C. \end{array}$$ (21)

Mouse experiments show that the tumor volume on average doubles within 5-15 days [21–23]. Assuming a linear growth

$$\begin{array}{c} \frac{dC}{dt}=\lambda_{0}C,\;\;\;\text{where}\;\lambda_{0}>0, \end{array}$$

during the volume doubling time, we conclude from Eq (21) that

$$\begin{array}{c} \lambda_{C}C(1-\frac{C}{C_{M}})-\left(\eta_{1}T_{1}+\eta_{8}T_{8}\right)C-d_{C}C=\lambda_{0}C. \end{array}$$ (22)

where $\lambda_{0}\in (\frac{\;\text{ln}2}{15},\frac{\;\text{ln}2}{5})$. We assume that with no immune responses

$$\begin{array}{c} \frac{dC}{dt}=2\lambda_{0}C, \end{array}$$

so that, by Eq (20), we get

$$\begin{array}{c} \lambda_{C}C(1-\frac{C}{C_{M}})-d_{C}C=2\lambda_{0}C. \end{array}$$ (23)

We take *λ*_0_ = 0.069/day, and assume that in steady state of Eqs (20) and (21), *C* is approximately 0.4 g/cm^3^, so that from Eq (23) we get

$$\begin{array}{c} \frac{1}{2}\lambda_{C}-d_{C}=2\lambda_{0}, \end{array}$$

or *λ*_*C*_ = 0.616/day. From Eqs (23) and (22), we get *η*_1_*T*_1_ + *η*_8_*T*_8_ = *λ*_0_. Noting that *T*_8_ cells kill cancer cells more effectively than *T*_1_ cells, we take *η*_8_ = 4*η*_1_, so that $\eta_{1}=\frac{\lambda_{0}}{T_{1}+4T_{8}}=11.5$ cm^3^/g ⋅ day and *η*_8_ = 46 cm^3^/g ⋅ day (with *T*_1_ = *K*_*T*_1__ = 2 × 10^−3^ g/cm^3^ and *T*_8_ = *K*_*T*_8__ = 1 × 10^−3^ g/cm^3^ as in the estimates for Eqs (3) and (4)).

Eq (6): With no drugs, the steady state plasma concentration of GM-CSF is $G={\hat{K}}_{G}=1.74\times 10^{-10}$ g/cm^3^ [60]. The half-life of GM-CSF is 13 hours [61], i.e, 0.54 day, so that $d_{G}=\frac{\;\text{ln}2}{0.54}=1.28$/day. From the steady state of Eq (6) (with no drugs), we get $\lambda_{G}=d_{G}G=d_{G}{\hat{K}}_{G}=2.23\times 10^{-10}$ g/cm^3^ ⋅ day. But when the GM-CSF-secreting vaccine is administered, the concentration of GM-CSF shoots up, and we assume that at steady states it reaches the level of $G=K_{G}=10{\hat{K}}_{G}=1.74\times 10^{-9}$ g/cm^3^.

Eq (7): From the steady state of Eq (7), we get *λ*_*I*_12_*D*_*D* − *d*_*I*_12__*I*_12_ = 0, where *d*_*I*_12__ = 1.38/day [59] and *I*_12_ = *K*_*I*_12__ = 1.5 × 10^−10^ g/cm^3^ [59], and *D* = *K*_*D*_ = 4 × 10^−4^ g/cm^3^. Hence, *λ*_*I*_12_*D*_ = 5.18 × 10^−7^/day.

Eq (8): From the steady state of Eq (8), we get *λ*_*I*_2_*T*_1__*T*_1_−*d*_*I*_2__*I*_2_ = 0, where *d*_*I*_2__ = 2.376/day [59] and *I*_2_ = *K*_*I*_2__ = 2.37 × 10^−11^ g/cm^3^ [59], and *T*_1_ = *K*_*T*_1__ = 2 × 10^−3^ g/cm^3^. Hence, *λ*_*I*_2_*T*_1__ = 2.82 × 10^−8^/day.

Eqs (9)–(12): In order to estimate the parameter *K*_*TQ*_ (in Eqs (3) and (4)), we need to determine the steady state concentrations of *P* and *L* in the control case (no drugs). To do that, we need to estimate *ρ*_*P*_ and *ρ*_*L*_. By [62], the mass of one PD-1 protein is *m*_*P*_ = 8.3 × 10^−8^ pg = 8.3 × 10^−20^ g, and by [63] the mass of one PD-L1 is *m*_*L*_ = 5.8 × 10^−8^ pg = 5.8 × 10^−20^ g. We assume that the mass of one T cell is *m*_*T*_ = 10^−9^ g. By [14], there are 3000 PD-1 proteins and 9000 PD-L1 proteins on one T cell (*T*_1_ or *T*_8_). Since *ρ*_*P*_*T* is the density of PD-1 (without anti-PD-1 drug), we get $\rho_{P}=3000\times \frac{m_{P}}{m_{T}}=\frac{3000\times (8.3\times 10^{-20})}{10^{-9}}=2.49\times 10^{-7}$ and $\rho_{L}=9000\times \frac{m_{L}}{m_{T}}=\frac{9000\times (5.8\times 10^{-20})}{10^{-9}}=5.22\times 10^{-7}$.

In steady state, *T*_1_ = 2 × 10^−3^ g/cm^3^ and *T*_8_ = 1 × 10^−3^ g/cm^3^. Hence, in steady state,

$$\begin{array}{ccc} P & = & \rho_{P}\left(T_{1}+T_{8}\right)=\left(2.49\times 10^{-7}\right)\times \left(2\times 10^{-3}+1\times 10^{-3}\right)=7.47\times 10^{-10}\;\;\text{g}/\;{\text{cm}}^{3}. \end{array}$$

The parameter *ε* in Eq (10) depends on the type of cancer; many cancer cells, but not all, express PD-L1 [17, 35–37]. Accordingly, we assume *ε* varies in the interval 0-0.01, but in the simulations we take *ε* = 0.01. Then, from Eq (10), we get

$$\begin{array}{ccc} L & = & \rho_{L}\left(T_{1}+T_{8}+\varepsilon C\right)=\left(5.22\times 10^{-7}\right)\times \left[2\times 10^{-3}+1\times 10^{-3}+0.01\times 0.4\right]\\ & = & 3.654\times 10^{-9}\;\text{g}/\;{\text{cm}}^{3}. \end{array}$$

In steady state with $P=\bar{P}$, $L=\bar{L}$ and $Q=\bar{Q}$, we have, by Eq (12), $\bar{Q}=\sigma \bar{P}\bar{L}$. We take $K_{TQ}=\frac{1}{2}\bar{Q}=\frac{1}{2}\sigma \bar{P}\bar{L}$. Hence, $Q/K_{TQ}=PL/\left(\frac{1}{2}\bar{P}\bar{L}\right)$ and

$$\begin{array}{c} \frac{1}{1+Q/K_{TQ}}=\frac{1}{1+PL/(\frac{1}{2}\bar{P}\bar{L})}=\frac{1}{1+PL/K_{TQ}^{\prime }}, \end{array}$$

where $K_{TQ}^{\prime }:=\frac{1}{2}\bar{P}\bar{L}=\frac{1}{2}\times \left(7.47\times 10^{-10}\right)\times \left(3.654\times 10^{-9}\right)=1.365\times 10^{-18}$ g^2^/cm^6^.

Eq (13): By [42], the half-life of anti-PD-1 is 15 days, so that $d_{A}=\frac{\;\text{ln}2}{15}=4.62\times 10^{-2}$ day^−1^. We assume that 10% of A is used in blocking PD-1, while the remaining 90% degrades naturally. Hence, in steady state, *μ*_*PA*_*PA*/10% = *d*_*A*_*A*/90%, so that

$$\begin{array}{c} \mu_{PA}=\frac{d_{A}}{9P}=\frac{4.62\times 10^{-2}}{9\times (7.47\times 10^{-10})}=6.87\times 10^{6}\;\;{\text{cm}}^{3}/\;\text{g}\cdot \;\text{day}. \end{array}$$

If the percentage of *A* used in blocking PD-1 is changed, the value of *μ*_*PA*_ will also change, but the results of simulations do not change qualitatively (not shown here).

### Sensitivity analysis

**Fig 8:**
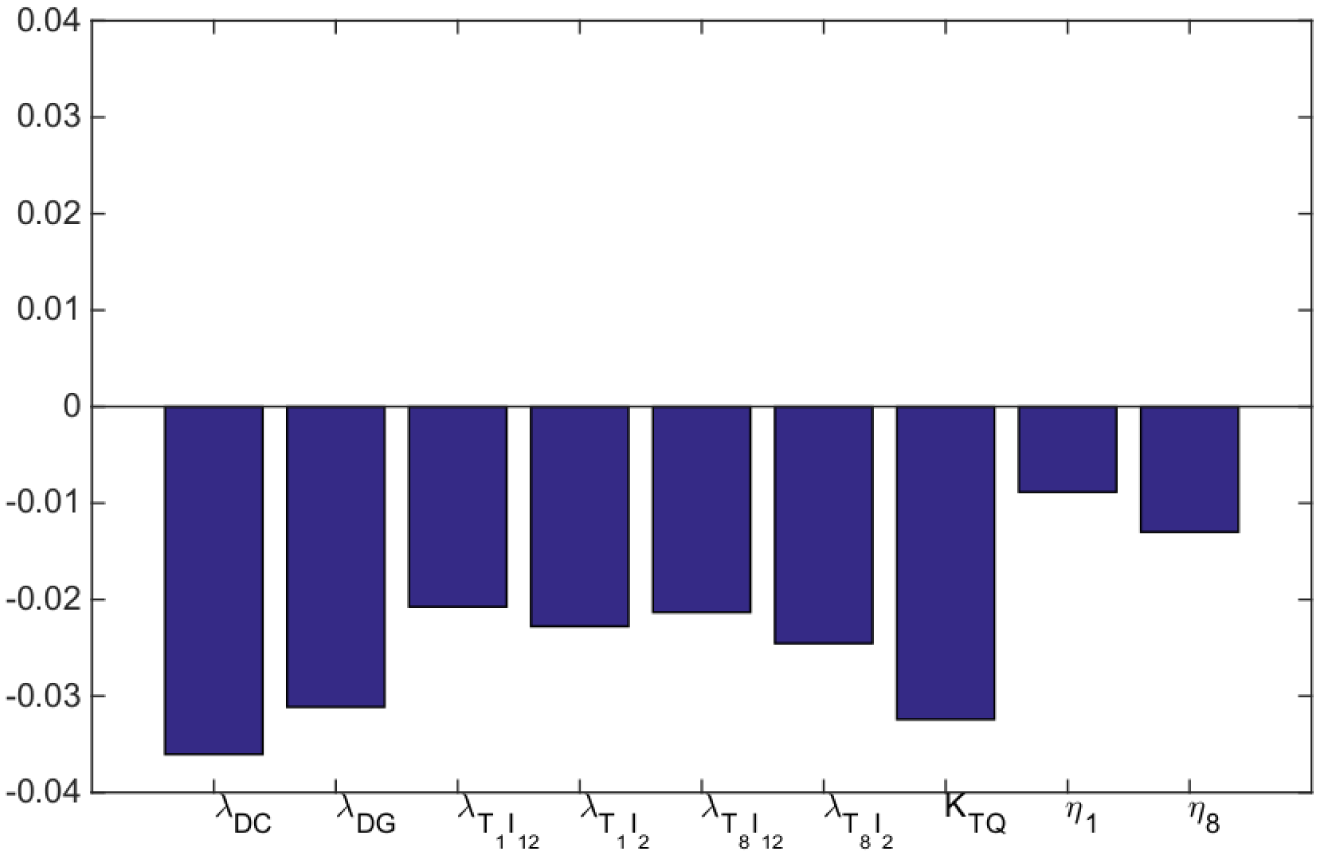
Statistically significant PRCC values (p-value < 0.01) for *R*(*t*) at day 60.

We performed sensitivity analysis, with respect to the tumor radius *R* at day 60, with respect to some of the production parameters of the System (2)–(13), namely, *λ*_*DC*_, *λ*_*DG*_, *λ*_*T*_1_*I*_12__, *λ*_*T*_1_*I*_2__, *λ*_*T*_8_*I*_12__, *λ*_*T*_8_*I*_2__, and the important parameters *K*_*TQ*_, *η*_1_ and *η*_8_. Following the method in [64], we performed Latin hypercube sampling and generated 1000 samples to calculate the partial rank correlation coefficients (PRCC) and the p-values with respect to the tumor radius at day 60. In sampling all the parameters, we took the range of each from 1/2 to twice its values in Tables 2 and 3. The results are shown in Fig 8.

Not surprisingly all the parameters are negatively correlated with the tumor radius. We note that the highest negatively correlated parameters are the activation rate of dendritic cells by cancer cells (*λ*_*DC*_) and the inhibition of T cells activation by PD-1-PD-L1 complex (*K*_*TQ*_). However, with different values of *γ*_*G*_ and *γ*_*A*_ the parameter *λ*_*DG*_ can exceed *λ*_*DC*_.

### Computational method

We employ moving mesh method [34] to numerically solve the free boundary problem for the tumor proliferation model. To illustrate this method, we take Eq (2) as example and rewrite it as the following form:

$$\begin{array}{c} \frac{\partial D(r,t)}{\partial t}=\delta_{D}\Delta D\left(r,t\right)-div\left(uD\right)+F,\; \end{array}$$ (24)

where *F* represents the term in the right hand side of Eq (2). Let $r_{i}^{k}$ and $D_{i}^{k}$ denote numerical approximations of i-th grid point and $D(r_{i}^{k},n\tau )$, respectively, where *τ* is the size of time-step. The discretization of Eq (24) is derived by the fully implicit finite difference scheme:

$$\begin{array}{c} \frac{D_{i}^{k+1}-D_{i}^{k}}{\tau }=\delta_{D}(D_{rr}+\frac{2}{r_{i}^{k}}D_{r})-(\frac{2}{r_{i}^{k+1}}u_{i}^{k+1}+u_{r})D_{i}^{k+1}-u_{i}^{k+1}D_{r}+F_{i}^{k+1}, \end{array}$$ (25)

where $D_{r}=\frac{h_{-1}^{2}D_{i+1}^{k+1}-h_{1}^{2}D_{i-1}^{k+1}-\left(h_{1}^{2}-h_{-1}^{2}\right)D_{i}^{k+1}}{h_{1}\left(h_{-1}^{2}-h_{1}h_{-1}\right)}$, $D_{rr}=2\frac{h_{-1}D_{i+1}^{k+1}-h_{1}D_{i-1}^{k+1}+\left(h_{1}-h_{-1}\right)D_{i}^{k+1}}{h_{1}\left(h_{1}h_{-1}-h_{-1}^{2}\right)}$, $u_{r}=\frac{h_{-1}^{2}u_{i+1}^{k+1}-h_{1}^{2}u_{i-1}^{k+1}-\left(h_{1}^{2}-h_{-1}^{2}\right)u_{i}^{k+1}}{h_{1}\left(h_{-1}^{2}-h_{1}h_{-1}\right)}$, $h_{-1}=r_{i-1}^{k+1}-r_{i}^{k+1}$ and $h_{1}=r_{i+1}^{k+1}-r_{i}^{k+1}$. The mesh moves by $r_{i}^{k+1}=r_{i}^{k}+u_{i}^{k+1}\tau$, where $u_{i}^{k+1}$ is solved by the velocity equation.
